# Establishment of Surgical Difficulty Grading System and Application of MRI-Based Artificial Intelligence to Stratify Difficulty in Laparoscopic Rectal Surgery

**DOI:** 10.3390/bioengineering10040468

**Published:** 2023-04-12

**Authors:** Zhen Sun, Wenyun Hou, Weimin Liu, Jingjuan Liu, Kexuan Li, Bin Wu, Guole Lin, Huadan Xue, Junjun Pan, Yi Xiao

**Affiliations:** 1 Division of Colorectal Surgery, Department of General Surgery, Peking Union Medical College Hospital, Chinese Academy of Medical Sciences and Peking Union Medical College, No. 1 Shuai Fu Yuan, Dongcheng District, Beijing 100730, China; 2 Department of Colorectal Surgery, National Cancer Center, National Clinical Research Center for Cancer, Cancer Hospital, Chinese Academy of Medical Sciences and Peking Union Medical College, Beijing 100021, China; 3 State Key Laboratory of Virtual Reality Technology and Systems, Beihang University, No. 37 Xueyuan Road, Haidian District, Beijing 100191, China; 4 Department of Radiology, Peking Union Medical College Hospital, Chinese Academy of Medical Sciences and Peking Union Medical College, Beijing 100730, China; 5 Peng Cheng Laboratory, No. 2 Xingke 1st Street, Nanshan District, Shenzhen 518055, China

**Keywords:** artificial intelligence, MRI, laparoscopic rectal surgery, surgical difficulty, pelvis

## Abstract

(1) Background: The difficulty of pelvic operation is greatly affected by anatomical constraints. Defining this difficulty and assessing it based on conventional methods has some limitations. Artificial intelligence (AI) has enabled rapid advances in surgery, but its role in assessing the difficulty of laparoscopic rectal surgery is unclear. This study aimed to establish a difficulty grading system to assess the difficulty of laparoscopic rectal surgery, as well as utilize this system to evaluate the reliability of pelvis-induced difficulties described by MRI-based AI. (2) Methods: Patients who underwent laparoscopic rectal surgery from March 2019 to October 2022 were included, and were divided into a non-difficult group and difficult group. This study was divided into two stages. In the first stage, a difficulty grading system was developed and proposed to assess the surgical difficulty caused by the pelvis. In the second stage, AI was used to build a model, and the ability of the model to stratify the difficulty of surgery was evaluated at this stage, based on the results of the first stage; (3) Results: Among the 108 enrolled patients, 53 patients (49.1%) were in the difficult group. Compared to the non-difficult group, there were longer operation times, more blood loss, higher rates of anastomotic leaks, and poorer specimen quality in the difficult group. In the second stage, after training and testing, the average accuracy of the four-fold cross validation models on the test set was 0.830, and the accuracy of the merged AI model was 0.800, the precision was 0.786, the specificity was 0.750, the recall was 0.846, the F1-score was 0.815, the area under the receiver operating curve was 0.78 and the average precision was 0.69; (4) Conclusions: This study successfully proposed a feasible grading system for surgery difficulty and developed a predictive model with reasonable accuracy using AI, which can assist surgeons in determining surgical difficulty and in choosing the optimal surgical approach for rectal cancer patients with a structurally difficult pelvis.

## 1. Introduction

The standard surgical treatment for rectal cancer is total mesorectal excision (TME). However, laparoscopic TME is challenging, due to the narrow and deep confines of the pelvic cavity [1]. There is no widely accepted definition of the “difficult pelvis”, however, by surgeons across the board [2]. Recently, there has been an increasing interest in factors affecting the difficulty of performing surgery in the pelvic cavity. Several studies have shown that the tumor location, tumor size, gender, body mass index (BMI), pelvic dimensions and angles, previous abdominal surgery, and neoadjuvant radiotherapy affect this difficulty [3,4,5,6]. However, these objective indicators are unreliable and, may not able to reflect the intraoperative situation. Meanwhile, transanal total mesorectal excision (taTME) and robotic surgery have offered solutions for patients with a technical difficulty in pelvic dissection [7,8]. Hence, a reliable indicator of difficulty with adaptable criteria may help to decide the optimal surgical approach.

MRI plays an important role in the management of rectal cancer [9]. Going a step further, it could provide more useful radiomics signatures that have not been exploited by traditional approaches. Artificial intelligence (AI) leverages computer algorithms to learn from data, extract features, help identify patterns in data, and make predictions, which shows great application prospects in medical research [10]. Previous studies have reported that AI has been utilized to guide decision-making in clinical practices, especially in evaluating cancer stage and response to therapy [11,12,13]. Additionally, it could guide surgeons during operation by analyzing intraoperative images [1,14]. In addition to these practices, there is progress in applying these advancements in AI technology to minimally invasive surgical procedures.

Considering the above factors, this study aimed to establish a reasonable grading system for surgical difficulty and to investigate the applicability and advancement of AI in evaluating the difficulty of laparoscopic rectal surgery, in terms of the pelvis.

## 2. Materials and Methods

**Figure 1:**
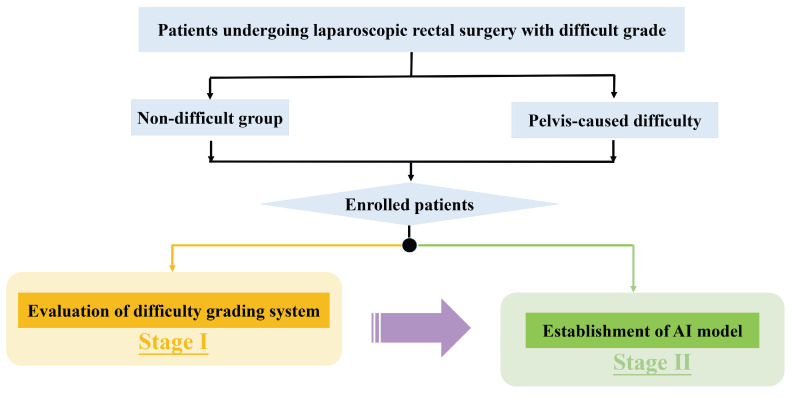
Research flowchart (Stage I: evaluating the difficulty grading system by comparing the perioperative outcomes of patients in the non-difficult group and the difficult group; Stage II: establishing an AI model to stratify surgical difficulty preoperatively).

There were two stages in this study (Figure 1). Perioperative characteristics of the difficult group and non-difficult group patients were compared to evaluate the feasibility of the surgical difficulty grading system in the first stage. In the second stage, patients were split into training set, validation set and test set (the proportions of difficult patients in the three sets were comparable). MR images and clinical variables, including BMI, gender and neoadjuvant information, were used to establish the difficulty prediction model. The primary outcome measure was the performance of the model.

### 2.1. Patients

**Figure 2:**
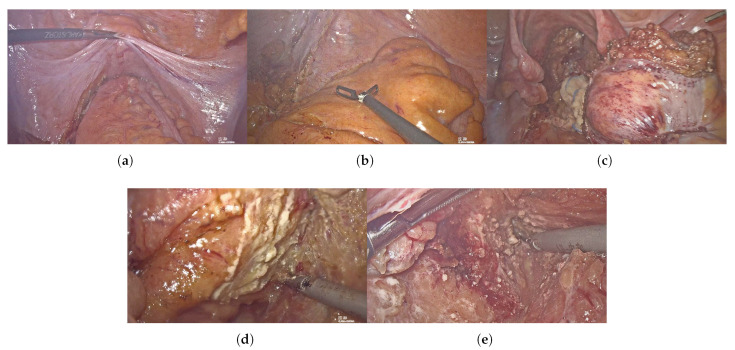
Visualization of the reasons for surgical difficulty (**a**) narrow pelvis; (**b**) thick mesorectum; (**c**) large tumor size; (**d**) tissue edema; (**e**) indistinct anatomical layer.

Data were collected from a prospectively established rectal cancer database in the Division of Colorectal Surgery, Department of General Surgery, Peking Union Medical College Hospital. Only patients in the database who had been graded according to the surgical difficulty system (Table 1) were enrolled in this study, from March 2019 to October 2022. The surgical difficulty grading system was established according to the surgeon’s experience and specimen quality. Surgeons were asked to evaluate and grade the difficulty of surgery basing on the following reason: 1. narrow pelvis; 2. thick mesorectum; 3. large tumor size; 4. tissue edema after radiotherapy; 5. indistinct anatomical layer. These reasons are visualized in Figure 2, and the demonstration video of each difficulty grade is listed in the Supplementary Material.

Graded patients were eventually enrolled in this study, based on the inclusion criteria of 1. a tumor within 12 cm from the anal verge; 2. preoperative MRI was accessible; 3. the depth of tumor invasion was $T_{1-4a}$. In this study, we focused on analyzing surgical difficulties caused by pelvic structures, and difficulties caused by other reasons have not been included so far. Grade I represents no surgical difficulty (non-difficult group), whereas grade II-IV represented patients with surgical difficulty (difficult group).

The protocol was designed according to the Transparent Reporting of a multivariable prediction model for Individual Prognosis or Diagnosis (TRIPOD) [15], approved by the Ethics Committee of Peking Union Medical College Hospital (No. S-K1585) and registered with the Chinese Clinical Trial Registry (ChiCTR2200059831). Written informed consent was obtained.

### 2.2. Image Preprocessing

In stage I, perioperative outcomes were aligned to the graded surgical difficulty. We consequently started the next phase by tagging non-difficult group patients with the label 0, and difficult group patients with the label 1. Therefore, the whole task could be transformed into a binary classification task (difficult and non-difficult) at stage II.

**Figure 3:**
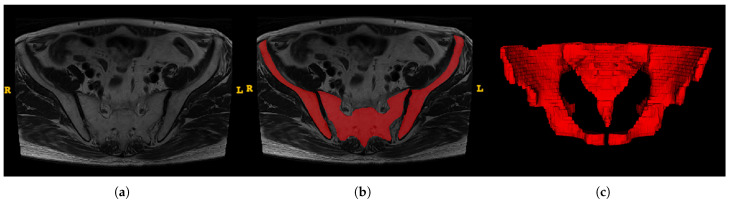
An example of manual segmentation of pelvis in MRI. (**a**) Bilateral ilium and sacrococcyx in T2-weighted images; (**b**) Manual segmentation on the same axial slice (Bilateral ilium and sacrococcyx are highlighted in red); (**c**) reconstruction of the pelvis.

ITK-SNAP software (version 3.8.0, www.itksnap.org) was utilized to obtain the region of interest (ROI) for further analysis [16]. The ROIs were carefully delineated along with the pelvis (Figure 3). Two trained surgeons (Z.S., W.Y.H.) independently conducted segmentation, and two experienced radiologists (J.J.L., H.D.X.) were introduced for supervision.

To normalize the different scan settings between MR images of patients, the images and annotations were resampled to 1.5 mm×1.5 mm×1.5 mm voxel spacing using the SimpleITK library [17,18], and were then converted to the Neuroimaging Informatics Technology Initiative (NIfTI) format. To avoid the interference of the grayscale texture and irrelevant tissue, and for the the neural network model to pay attention to the bone structure and pelvic distance within the ROI, we set the voxel value to 0 for the non-ROI area, and to 1 for the bilateral ilium and the sacrococcyx. All images are padded to the same size, with a value of 0.

#### 2.2.1. Network Architecture

**Figure 4:**
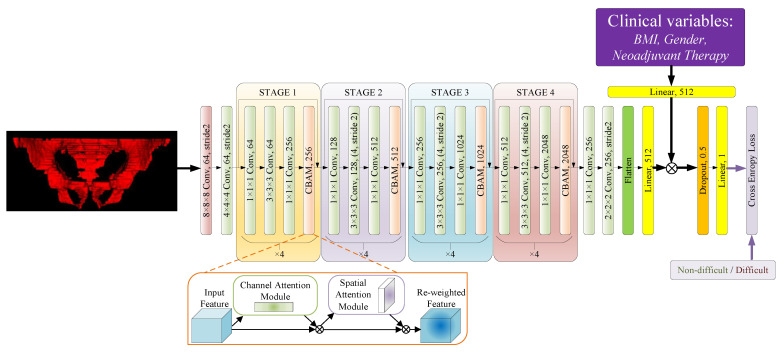
MRI image and clinical variables were used for feature extraction by the proposed modified ResNet-50 (⨂ means element-wise multiplication) and the cross-entropy loss in Equation (2) was adopted for supervision.

Since the data structure and distribution of the images were not complicated, the widely used ResNet-50 network [19] was chosen as the basic backbone model for this classification task, and a Convolutional Block Attention Module (CBAM) [20] was added to each ResNet block to improve the ability of the model to pay more attention to the spatial and feature relationships. The CBAM module contains both channel attention and spatial attention blocks. The channel attention block helps the network to re-weight the channels of the features and focus more on the important channels. Additionally, the spatial attention mechanism is helpful in this spatial task, by forcing the network to pay more attention to the important regions, which can further improve the the presentation of grade-based heat map calculation described below. The classifiers in the network were modified with additional linear and dropout layers, to avoid the risk of overfitting and to improve the convergence ability of the network model, and the clinical variables consisting of BMI, gender and neoadjuvant therapy were nomalized to (0,1), followed by the addition of a linear layer to re-weight the features captured by the model for the final classification. Such variables were adopted because, according to our research described below, they correlate with the surgical difficulty, but can not be easily achieved from the pelvic bone structure. Another modification was that we replaced all the pooling layers with convolution layers, and the last global average pooling layers were replaced by a combination of a shrink convolution layer and a 2-stride downsample convolution layer, in order to force the network to gather the feature information at the correlative position. Besides, all the convolution layers with a stride of 2 were changed to an even kernel size to avoid position offsets during the downsampling process. The structure of the proposed modified ResNet-50 network is shown in Figure 4. and the hyper-parameters and details of the network are shown in the Supplementary Material and Appendix A Table A1.

#### 2.2.2. Data Augmentation

In order to improve the generalizability of the network and the diversity of the dataset, and to reduce the risk of overfitting, random augmentation [21] was applied to the training process. To ensure the consistency of the spatial relationship, random left–right flips and $\left[-5^{\circ },+5^{\circ }\right]$ max random axial rotations around the center were adopted. For each patient per epoch in the training set, random augmentation was performed and enlarged 4 times. The probability of random left–right flipping was set to 0.5, and the probability of random axial rotation was set to 0.4.

#### 2.2.3. Implementation and Metrics

The deep neural network models were implemented using the PyTorch framework (version 1.12.1, pytorch.org, accessed on 6 August 2022). The cross-entropy loss is the loss function during the training process, which is formulated as follows:

(1) $$argmin_{x}L\left(x\right)=-\left[y\cdot logf\left(x\right)+\left(1-y\right)\cdot log\left(1-f\left(x\right)\right)\right],$$

where *x* means the input volume and *y* means the ground truth of the case. Function f(·) represents the model pipeline, followed by a sigmoid function.

AdamW optimizers were applied with the weight decay set to $1\times 10^{-8}$. The learning rate was set to $1\times 10^{-6}$. The model was trained with the batch-size set to 4, for at least 7000 iterations and the gradient-clipping method was set to 0.5; the early-stop method was used in the validation set to avoid the risk of overfitting.

### 2.3. Feature Extraction and Model Construction

**Figure 5:**
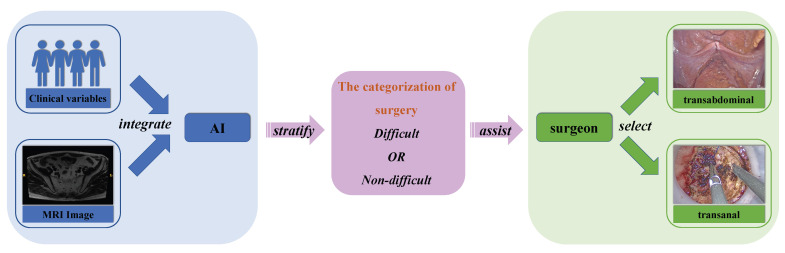
Overview of the AI model Process.

Segmented MRI images and clinical variables, including BMI, gender and neoadjuvant therapy information, were retrospectively retrieved for feature extraction and evaluation. After feature evaluation, useful signatures were integrated to develop the model, which would preoperatively stratify the difficulty of pelvic operation, and assist surgeons in selecting optimal surgical approaches (Figure 5).

### 2.4. Visualization of the Attention Region

**Figure 6:**
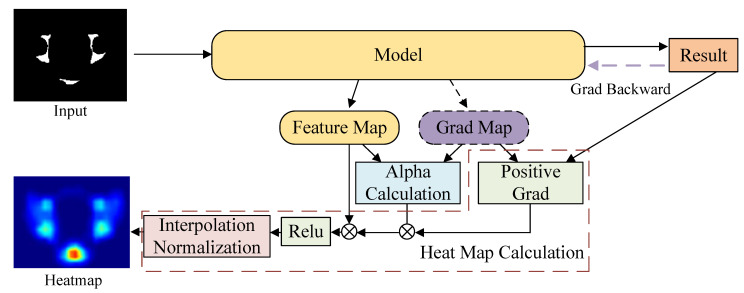
Visualization of the attention region of model by GradCAM++. The heatmap is generated based on the Equation (2) (Alpha Calculation) and Equation (3) (heat map calculation).

The Class Activation Map (CAM) [22,23] can provide visible evaluation of the attention region of the neural network model, in order to make a class judgment based on the prediction results and the gradient. To fully visualize the process during the inference of the model, we used GradCAM++ [24], one of the most popular CAM methods, in our experiments. The computational process of GradCAM++ is shown in Figure 6, which is formulated as follows:

(2) $$\alpha =\frac{g^{2}}{R_{0\to 1}\left(g^{2}+S_{xyz}\left(f\cdot g^{3}\right)\right)},$$

(3) $$heatmap=S_{c}\left(S_{xyz}\left(\alpha \cdot Relu\left(g\right)\right)\cdot f\right),$$

where *f* and *g* are the feature and gradient maps of the selected layer, respectively,0 and $R_{0\to 1}\left(\cdot \right)$ replaces zeros in the inputs with ones. $S_{c}$ and $S_{xyz}$ are the sum functions along the feature and space channels.

For adapting the traditional 2D CAM technique to the 3D data, the 2D calculations were replaced with 3D ones, and then a 3D heatmap with the same size of the input was obtained. Results could be fully visible using JET color space in the ITK-SNAP software. In order to comprehensively explore the attention distribution for the skeletal region, the Demons non-rigid registration method [25] implemented in the SimpleITK library was applied, in order to unify all the data inputs and heatmap within the same space. Then, the averaged skeletal structure and the corresponding average heatmap could be obtained, with calculations of the average values.

### 2.5. Statistics Analysis

Clinical information was analyzed using SPSS version 26.0 (IBM SPSS INC., Chicago, IL, USA). Continuous data were shown as medians with interquartile ranges (IQR) or means with standard deviations (SD). Categorical data were shown as numbers with percentages. Continuous variables were compared using Mann-Whitney test or independent *t* test and the Chi-squared test was used for the comparison of categorical variables. p<0.05 was considered statistically significant.

Several metrics consisting of accuracy, specificity, precision, recall, and F1-score were used to evaluate the network prediction results and network prediction ability [26].

## 3. Results

### 3.1. Grouped Patient Characteristics

Among the 108 enrolled patients, the median age was 64 (56–70) years, and 73 patients were male. The mean BMI was 24.1 ± 3.4 kg/m ${}^{2}$, the mean distance between the lower edge of the tumor and the anal verge was 6.5 ± 2.0 cm and the median tumor size was 2.0 (1.3–3.1) cm. A total of 77 patients received neoadjuvant therapy. And 13 patients had a history of abdominal surgery. Patients were divided into the difficult group (n = 53) and non-difficult group (n = 55), as shown in Table 2.

### 3.2. Associations of Perioperative Outcomes with Surgical Difficulty (Stage I)

Considering there was no anastomosis in extralevator abdominoperineal excision (ELAPE) and Hartmann procedures, six patients were excluded in stage I. In total, 48 difficult patients (Grade II-IV) and 54 non-difficult patients (Grade I) were compared to evaluate the feasibility of the surgical difficulty grading system. As shown in Table 3, the proportions of male patients and those receiving neoadjuvant chemoradiotherapy in the difficult group were larger than in the non-difficult group. The mean BMI was larger in the difficult group. The duration of surgery and blood loss showed significant increases in the difficult group. What is more, the proportions of complete TME, diverting stoma and anastomotic leaks were larger in the difficult group. However, the lymph nodes harvested, postoperative complications and postoperative hospital stays were comparable between two groups.

### 3.3. The Performance of Model (Stage II)

#### 3.3.1. Cross Validation Study

To fully validate the performance and generalization of the proposed model, we performed a 4-fold cross validation experiment based on the training dataset. The datasets for each fold consist of images of 63 patients for training, and images of 20 patients for validation. The final accuracy, precision, specificity, recall and F1 score results in each fold are presented with the average of each metric in Table 4. The result shows that the model can achieve good performance with sufficient generalization.

#### 3.3.2. The Performance of the Merged Model

**Figure 7:**
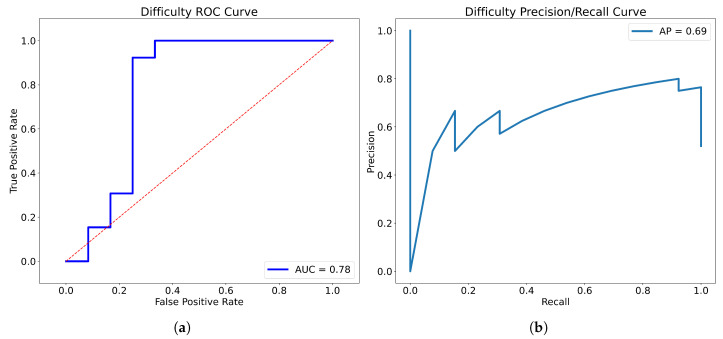
The ROC curve (**a**) and P-R curve (**b**) of the merged model.

**Figure 8:**
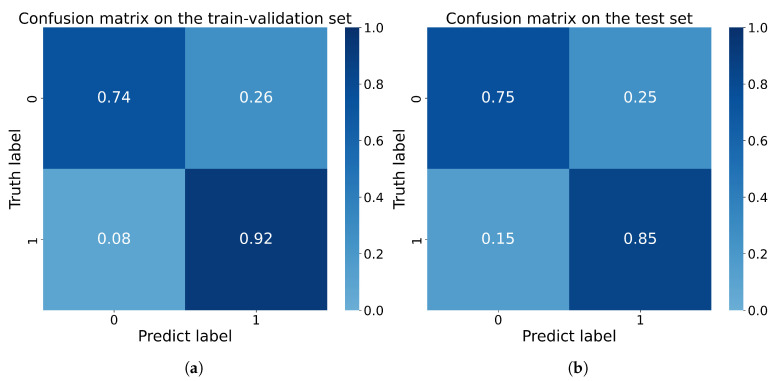
The confusion matrix of (**a**) the train-validation set and (**b**) test set based on the merged model.

**Figure 9:**
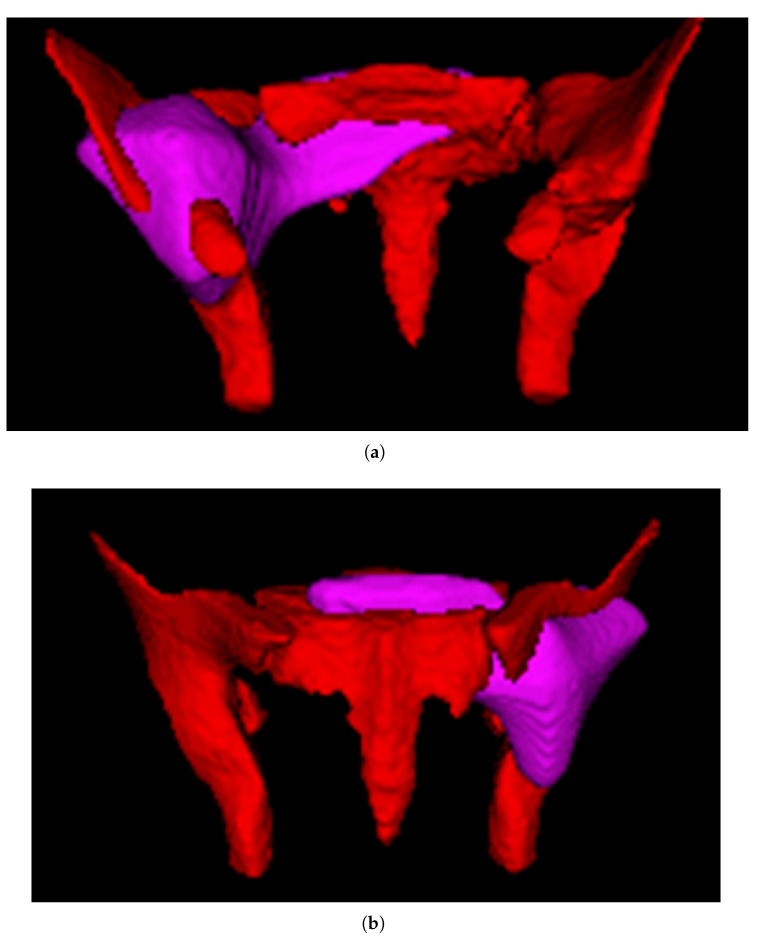
Highlighted heatmap of the attention region. (**a**) anterior view. (**b**) posterior view.

After training, we merged the 4-fold models used above by averaging the prediction scores, and tested the merged model on the separate test set, consisting of images of 25 patients unused in the training process; the results of each fold on the test set are presented in Table 5. The accuracy of the model in terms of the test set was 0.800, the precision of the model was 0.786, and the specificity, recall, and F1 score were 0.750, 0.846, and 0.815, respectively. The Receiver Operating Characteristic (ROC) curve and Precision-Recall (P-R) curve are shown in Figure 7, the Area Under Curve (AUC) was 0.78 and the Average Precision (AP) was 0.69. The confusion matrix results of the merged model in the training set and test set are shown in Figure 8. Then, all the 4-fold established models were taken for the subsequent experiments to generate the visible heatmaps of the region’s attention. In Figure 9, after the non-rigid registration process, we took the mean results of the heatmap generated from layer 4 in the network, and voxels with the top 64,000 (around 0.5%) heat scores were highlighted. As shown in Figure 9, the purple region indicates the concentration of factors causing difficulty.

## 4. Discussion

The significance of this study is that it brings a new concept to the standard clinical procedure of preoperative assessment of the optimal surgical options for rectal cancer patients. It also attempts to define, stratify, and quantify difficulty levels to direct the surgeon’s practice. This study showed that the grading system could stratify patients who underwent rectal surgery into different categories of difficulty status, namely non-difficult and difficult. Compared with the non-difficult group, the duration of surgery was longer, intraoperative blood loss was greater, the quality of specimens was poorer and the proportions of diverting stoma and anastomotic leaks were higher in the difficult group. What is more, this study firstly demonstrated that AI could stratify the pelvic difficulty of laparoscopic rectal surgery with good performance by incorporating radiomics and clinical features—the area under the receiver operating curve was 0.78 and the average precision was 0.69. The difficulty grading system and AI model could enable the concept of the individualized surgical management of patients with rectal cancer.

The incidence of anastomotic leaks and overall survival largely depend on surgical quality [27,28]; there is an increasing interest in exploring the factors that affect the difficulty of laparoscopic rectal surgery [3,29,30] The first attempt of colorectal surgeons was to define and classify difficult surgery. Previous studies have chosen the following criteria to define surgical difficulty: duration of surgery [6,31,32,33], blood loss [32], conversion to open surgery, the incidence of morbidity [34], and quality of surgery [35]. The risk of these criteria is that some criteria could be affected by various factors, including the surgeon’s skills, tumor location, and patient’s condition. Pure objective indicators are not included, and hence may not able to reflect the intraoperative situation. We believe intraoperative scoring by trained surgeons and the quality of specimen would result in a more accurate measurement for the judgment of difficulty. Thus, a difficulty grading system based on the surgeon’s experience and specimen quality was firstly purposed by this study. A narrow pelvis, thick mesorectum, large tumor size, tissue edema, and indistinct anatomical layer were selected to account for the surgical difficulty in this grading system. In the narrow anatomical space of the deep pelvis, a larger tumor size and thicker mesorectum have adverse effects on operation [3,4], limiting the vision of laparoscopy and restricting the ability of surgeons to operate. Neoadjuvant radiotherapy reduces local recurrence and benefits survival [36], but it also results in severe tissue edema followed by fibrosis [37], which increases the difficulty of dissecting the mesorectum. Consistent with the above findings, this study found that the proportions of males and those receiving neoadjuvant chemoradiotherapy were larger in the difficult group. Surgical outcomes between non-difficult patients and difficult patients were compared, indicating that the surgical difficulty grading system is reliable.

Having the comparable clear division of difficulty, surgeons went a further step by attempting to predict surgical difficulty for the purpose of supporting clinical practice. A number of tools have been developed to assist decision-making, such as risk-class stratifications [30], nomograms [3], etc. As the most commonly developed method, the repeatability and practicability of nomograms were being doubted [38]. Several studies used bony measurements to assess the difficult pelvis; the parameters, however, varied among different studies, limiting the comparison and utility of models [2]. The clinical applicability of these methods remains unclear, because of their retrospective nature, small sample sizes, and inadequate validation of these studies. Thus, previous studies concerning surgical difficulty prediction require improvement.

AI, involving the integration of automatic extraction and analysis of image characteristics that are invisible to the naked eye, together with conventional technology, has shown excellent performance in preoperative decision-making. MRI image-based deep learning could evaluate circumferential resection margisn [39], pathological complete response to neoadjuvant chemoradiotherapy [13] and identify metastatic lymph nodes [40]. CT image-based AI could detect peritoneal carcinomatosis of colorectal cancer [41]. Moreover, AI could analyze real-time laparoscopic images to guide operation [1]. Until recently, there has been no research exploring the value of AI in distinguishing between degrees of surgical difficulty.

Up to now, the current preoperative assessment method for this task is mainly based on manual measurement. However, manual measurement has the problems of anatomical marker selection and poor consistency among the patients. The AI system is able to extract the distance information more effectively to obtain better results. This study made the first attempt to establish a difficulty-prediction model by using AI. Although the AI system cannot fully replace the manual measurements for now, it is able to provide at least an additional preliminary judgment for the surgery. The model has shown good accuracy and reliability, which has the potential to assist surgeons in judging the surgical difficulty preoperatively and in choosing the optimal surgical approach for patients with rectal cancer (Figure 5). MRI images and clinical characters subjected to this model would output a prediction result (difficult or non-difficult). For patients categorized as difficult, transanal approaches are supposed to be considered, since the transabdominal approach may not have the ability to mobilize and resect the rectum in the deep narrow pelvis of patients. In contrast, for patients analyzed as non-difficult, surgeons could perform transabdominal surgery with confidence. Remaining as one of the top fields of AI, the calculation process of the AI model cannot be fully demonstrated by traditional parameters. Fortunately, the output heatmap drawn by CAM could show the attention region of the model, which could be aligned, to some extent, in clinical practice. As shown in Figure 9, the purple highlighted region was the key attention region affecting (pelvic) difficulty, nd was used by the AI model to judge the difficulty of surgery. The shape of this area was irregular and extended to the ilia and sacrococcyx, indicating that these structures had adverse influences on difficulty. The ilium confined the approach of surgical instruments and limited the bilateral dissection of the rectum in the deep and narrow pelvic cavity, which was consistent with the intraoperative experience of the surgeon. In the meanwhile, the rectum lies close to the posterior surface of the prostate, which adds the difficulty of dissection in males. In conclusion, the algorithm we created in this study could automatically extract characteristics and consequently develop a reliable prediction model. Besides this, the results of the heat map results also showed that the network exhibits a focus on the position relationship between the hip and ilium, and further study could help us to better simplify and optimize the manual measurement method, which we are considering for further research in the future.

## 5. Limitation

However, as the preliminary exploration in this issue, our study has some unavoidable limitations. First, the difficulty of surgery is a complex task, so, we only focused on the pelvic structures as an initial attempt in this study. However, the influence of non-bone structures such as tumor and mesorectum remains unknown. Second, the small sample size is still a limitation for further research, and more samples are needed to meet the requirement of more reliable deep learning models to ensure the robustness and generalization. We believe that in the future, by enriching the data samples, other factors can also be integrated in the research. Third, the clinical utility is limited by the cumbersome drawing of ROIs. Efforts are being made to develop an automated segmentation system for future application. Fourth, as a single-center retrospective study, selection bias cannot be completely avoided. What is more, we think the performance of the 3D convolution neural network (CNN)-based network might be limited by the implicit expression of special information, which means 3D-CNN might not be the the most suitable framework for this task. So, even with a reasonable performance in this task, we still have plans to progress the space-based models, such as point cloud models and graph neural networks (GNN).

## 6. Conclusions

In conclusion, the surgical difficulty grading system we have established is rational and practicable. The AI model has good diagnostic performance on the preoperative stratification of difficult surgery in patients with rectal cancer. Therefore, AI, as a novel method for individualized difficulty pelvis prediction, has widely potential application in decision-making. For the next step, further investigation by larger prospective studies is needed to improve the reliability of the grading system, and more efforts are needed to validate the predictive performance of the AI model. 

## Figures and Tables

**Table 1:** The difficulty grading system.

Grade	Definition
I	Easy procedure, without difficulty
II	Difficult procedure, but no impact on specimen quality (complete TME)
III	Difficult procedure, with slight impact on specimen quality (near-complete TME)
IV	Very difficult procedure, with severe impact on specimen quality (incomplete TME)

**Table 2:** Characteristics of enrolled patients.

Variable	Enrolled Patients(n = 108)	Difficult Group (n = 53)	Non-Difficult Group (n = 55)
Age, years, [median (IQR)]	64 (56–70)	66 (58–70)	63 (53–69)
Male, n (%)	73 (67.6)	51 (96.2)	22 (40.0)
BMI, kg/m ${}^{2}$, [mean (SD)]	24.1 (3.4)	25.0 (3.4)	23.2 (3.1)
Neoadjuvant chemoradiotherapy, n (%)	77 (71.3)	45 (84.9)	32 (58.2)
Previous abdominal surgery, n (%)	13 (12.0)	5 (9.4)	8 (14.5)
Distance from tumor to anal verge, cm [mean (SD)]	6.5 (2.0)	6.3 (2.0)	6.7 (2.0)
Tumor size, cm [median (IQR)]	2.0 (1.3–3.1)	2.0 (1.2–3.1)	2.0 (1.3–3.1)
Surgery type			
LAR, n (%)	79 (73.1)	35 (66.0)	44 (85.5)
taTME, n (%)	18 (16.7)	12 (22.6)	6 (10.9)
ISR, n (%)	5 (4.6)	1 (1.9)	4 (7.3)
Others, n (%)	6 (5.6)	5 (9.4)	1 (1.8)

LAR: low anterior resection; taTME: transanal total mesorectal excision; ISR: intersphincteric resection; Others: extralevator abdominoperineal excision (ELAPE), Hartmann.

**Table 3:** Comparison of diffiult and non-difficult groups of patients.

Variable	Surgical Difficulty		*p* Value
	Difficult Group (n = 48)	Non-Difficult Group (n = 54)	
Male, n (%)	46 (95.8)	21 (38.9)	<0.001
BMI, kg/m ${}^{2}$, [mean (SD)]	25.2 (3.3)	23.2 (3.1)	0.002 ${}^{\circ }$
Neoadjuvant chemoradiotherapy	41 (85.4)	32 (59.3)	0.003
Previous abdominal surgery	4 (8.3)	7 (13.0)	0.452
Distance from tumor to anal verge, cm [mean (SD)]	6.5 (1.8)	6.7 (2.0)	0.437 ${}^{\circ }$
Tumor size, cm [median (IQR)]	1.7 (1.2–3.0)	2.0 (1.3–3.0)	0.526 *
Duration of surgery, min [median (IQR)]	145.0 (120.0–160.0)	118.5 (100.0–141.3)	0.001*
Blood loss, mL [median (IQR)]	25 (20–50)	20 (10–40)	0.004 *
Diverting stoma, n (%)	43 (89.6)	37 (68.5)	0.010
Complete TME, n (%)	31 (64.6)	54 (100)	<0.001
Lymph nodes harvested, n [median (IQR)]	12 (8–17)	13 (10–17)	0.665 *
Postoperative complications, n (%)	17 (35.4)	19 (35.2)	0.981
Anastomotic leak, n (%)	8 (16.7)	2 (3.7)	0.043
Postoperative hospital stays, days [median (IQR)]	7 (6–7)	6 (6–8)	0.478 *

* Mann-Whitney test; ${}^{\circ }$ independent *t* test.

**Table 4:** Results of the 4-fold cross validation study on the validation set.

Fold	Accuracy	Precision	Specificity	Recall	F1 Score
1	0.850	0.889	0.900	0.800	0.842
2	0.750	0.692	0.600	0.900	0.782
3	0.850	0.818	0.800	0.900	0.857
4	0.850	0.818	0.800	0.900	0.857
Average	0.825	0.804	0.775	0.875	0.835

**Table 5:** Results of the 4-fold models on the test set.

Fold	Accuracy	Precision	Specificity	Recall	F1 Score
1	0.840	0.800	0.750	0.923	0.857
2	0.880	0.813	0.750	1.000	0.897
3	0.800	0.786	0.750	0.846	0.815
4	0.800	0.786	0.750	0.846	0.815
Average	0.830	0.796	0.750	0.904	0.846
Merged	0.800	0.786	0.750	0.846	0.815

## Data Availability

The data presented in this study are available on request from the corresponding author. The data are not publicly available due to ethical concerns.

## Supplementary Materials

The following supporting information can be downloaded at: https://www.mdpi.com/article/10.3390/bioengineering10040468/s1.

## Appendix A

**Table A1:** Hyper-parameters and details of the proposed model.

Block Names	Output Size	Layers	
Conv1	$\frac{1}{2}$	8×8×8,64,stride2	
Conv2_x	$\frac{1}{4}$	4×4×4,64,stride2	
		$\left(\begin{array}{c} 1\times 1\times 1,64\\ 3\times 3\times 3,64\\ 1\times 1\times 1,256\\ CBAM,256 \end{array}\right)\times 3$	
Conv3_x	$\frac{1}{8}$	$\left(\begin{array}{c} 1\times 1\times 1,128\\ 4\times 4\times 4,128,stride\;2\\ 1\times 1\times 1,512\\ CBAM,512 \end{array}\right)\times 1,\left(\begin{array}{c} 1\times 1\times 1,128\\ 3\times 3\times 3,128\\ 1\times 1\times 1,512\\ CBAM,512 \end{array}\right)\times 3$	
Conv4_x	$\frac{1}{16}$	$\left(\begin{array}{c} 1\times 1\times 1,256\\ 4\times 4\times 4,256,stride\;2\\ 1\times 1\times 1,1024\\ CBAM,1024 \end{array}\right)\times 1,\left(\begin{array}{c} 1\times 1\times 1,256\\ 3\times 3\times 3,256\\ 1\times 1\times 1,1024\\ CBAM,1024 \end{array}\right)\times 3$	
Conv5_x	$\frac{1}{32}$	$\left(\begin{array}{c} 1\times 1\times 1,512\\ 4\times 4\times 4,512,stride\;2\\ 1\times 1\times 1,2048\\ CBAM,2048 \end{array}\right)\times 1,\left(\begin{array}{c} 1\times 1\times 1,512\\ 3\times 3\times 3,512\\ 1\times 1\times 1,2048\\ CBAM,2048 \end{array}\right)\times 3$	
Classifier	(Batch size, 1)	1×1×1,256, 2×2×2,256,stride2	Other Inputs Normalization (MinMax, Binary)
		Flatten, Linear, →512	Linear, 3→512, Sigmoid
		Channel-wise Multiply	
		DropOut, 0.5, Linear, 512→1	
