# Supplementary material for: Establishment of Surgical Difficulty Grading System and Application of MRI-Based Artificial Intelligence to Stratify Difficulty in Laparoscopic Rectal Surgery
Source: Bioengineering (Basel). 2023 Apr 12;10(4):468. doi: 10.3390/bioengineering10040468 (PMC10135707; doi:10.3390/bioengineering10040468)
Supplement: Supplementary file 1 [file bioengineering-10-00468-s001.zip › Transcription.pdf]

This is the demonstration video of each difficulty grade.

Grade I: Easy procedure, without difficulty.

Grade II: Difficult procedure, but no impact on specimen quality (complete TME).

Grade III: Difficult procedure, with slight impact on specimen quality (near-complete TME).

Grade IV: Very difficult procedure, with severe impact on specimen quality (incomplete TME).
